# Virtual patients reflecting the clinical reality of primary care – a useful tool to improve cultural competence

**DOI:** 10.1186/s12909-021-02701-z

**Published:** 2021-05-11

**Authors:** Erica Rothlind, Uno Fors, Helena Salminen, Per Wändell, Solvig Ekblad

**Affiliations:** 1 Cultural Medicine, Department of Learning, Informatics, Management and Ethics, Karolinska Institutet, Stockholm, Sweden; 2 Department of Computer and Systems Sciences, Stockholm University, Stockholm, Sweden; 3 Department of Neurobiology, Care Sciences and Society, Division of Family Medicine and Primary Care, Karolinska Institutet, Huddinge, Sweden; 4 Academic Primary Health Care Centre, Region Stockholm, Stockholm, Sweden

**Keywords:** Computer simulation, Medical education, General practice, Primary care, Culture, Qualitative research

## Abstract

**Background:**

Virtual patients are educational tools that may be described as case-based interactive computer simulations of clinical scenarios. In terms of learning outcomes, improved clinical reasoning skills and knowledge acquisition have been shown. For further exploring the role of virtual patients in medical education, a greater focus on context-specific cases, combined with suitable educational activities, has been suggested. A knowledge gap has been identified in cultural competence in primary care. As primary care physicians are often the main medical providers for patients with refugee backgrounds, they would probably benefit from improved training focusing on how to apply cultural competence in everyday work. Using virtual patient cases, as a complement to clinical training, may be one way forward. The aim of this study was therefore to explore a learner perspective on the educational use of a virtual patient system designed to contribute to training in cultural competence in a primary care context.

**Methods:**

Three virtual patient cases portraying patients with refugee backgrounds were developed. The cases addressed various issues and symptoms common in primary care consultations, while also incorporating intercultural aspects. The system also provided the informants with individualized feedback. Primary care physicians and medical students were invited to test the cases and participate in an interview about their experience. Data was analyzed using qualitative content analysis.

**Results:**

The analysis generated the theme *Virtual patients might help improve cultural competence in physicians and medical students by complementing knowledge gained through the informal curriculum.* Informants at different educational levels found it suitable as a tool for introducing the topic and for reflecting on one’s own consultations. It could also compensate for the predominant informal manner of learning cultural competence, described by the informants.

**Conclusions:**

Virtual patients could be useful for gaining cultural competence in a primary care context. Advantages that could benefit learners at both pre- and post-graduate levels are decreased dependence on the informal curriculum and being presented with an illustrative way of how cultural competence may be applied in the consultation.

**Supplementary Information:**

The online version contains supplementary material available at 10.1186/s12909-021-02701-z.

## Background

Intercultural consultations, which by definition involve communication across cultures, are often perceived by both physicians and patients as challenging [1–6]. Communicational and cultural issues may also provide obstacles to health care on equal terms [1–8]. A group particularly at risk of being negatively affected by health disparities is patients with refugee backgrounds [7, 8]. Since the primary care physician (PCP) is often the initial point of contact and the main health provider for these patients, increased cultural competence is needed [9–11].

This identified need for improved competence in intercultural health care is not new, but there is still discussion on how to attain this [12–14]. Cultural competence is often defined as ‘a set of congruent behaviors, attitudes, and policies that come together in a system, agency, or among professionals that enables effective work in cross-cultural situations’ [15]. One main goal is to improve equity and thereby reduce disparities in health care [16, 17]. Cultural competence is a complex concept and despite the ongoing debate on its appropriateness in medical education and health care, it is to date probably the most established concept, and will therefore be used in this article [9, 14, 18]. Cultural competence is often a part of the informal curriculum in post-graduate education in primary care; with learning taking place experientially in the workplace and formal learning activities being scarce [9, 19–21]. Meanwhile, studies have indicated that resident physicians often perceive intercultural consultations as challenging, due to lack of both confidence and skills [22–24], and there has been an increased demand for education in cultural competence [16, 25]. Various teaching methods have been applied [9, 25] and the use of virtual learning environments, including virtual patients (VPs), has shown some promise [26–32].

Virtual patients (VPs) are educational tools used in medical education since the 1990s [33–35]. They are often described as “case-based interactive computer simulations of clinical scenarios” [33]. Their use in medical education is underpinned by experiential learning theory, with its theoretical model of action and reflection [36]. They have been used on pre- and postgraduate levels, using various educational set-ups (including problem-based learning), and in different areas of medical education, including cultural competence training, in both primary care and psychiatry [32, 37, 38].

The positive effect of VPs as learning activities on learning outcomes, such as knowledge and clinical reasoning, compared with no intervention, has already been established [26, 39, 40]. Compared to more passive forms of educational methods, learners may also benefit, especially if the intended learning objective is focused on clinical reasoning [35]. However, the quality of evidence is under discussion [35]. Media comparative research has been criticized for not contributing to the field of digital health education, as many confounding factors often invalidate control groups [41, 42]. Moreover, a shift in focus, from viewing VPs as software tools, to exploring their use as part of an educational activity, has been advocated [43, 44]. Exploring the possibility of making VPs more context-specific, in order to meet the needs of various medical specialties, has also been suggested [43]. Relevant aspects in a primary care context could, for example, be an emphasis on social determinants of health, or diagnostic reasoning of PCPs when dealing with multimorbidity or intercultural aspects [43, 45, 46]. Therefore, the present aim was to explore a learner perspective on the educational use of a VP system designed to contribute to cultural competence training in a primary care context.

## Methods

We chose a qualitative approach as this is an appropriate method for exploring views and experiences in a relatively unexplored area of research [47].

### Study design

#### Designing the VP system

The VP system used is called BSA-sim and is a further development of earlier VP systems, such as Web-SP from Karolinska Institutet and Stockholm University [30–32].

**Fig. 1:**
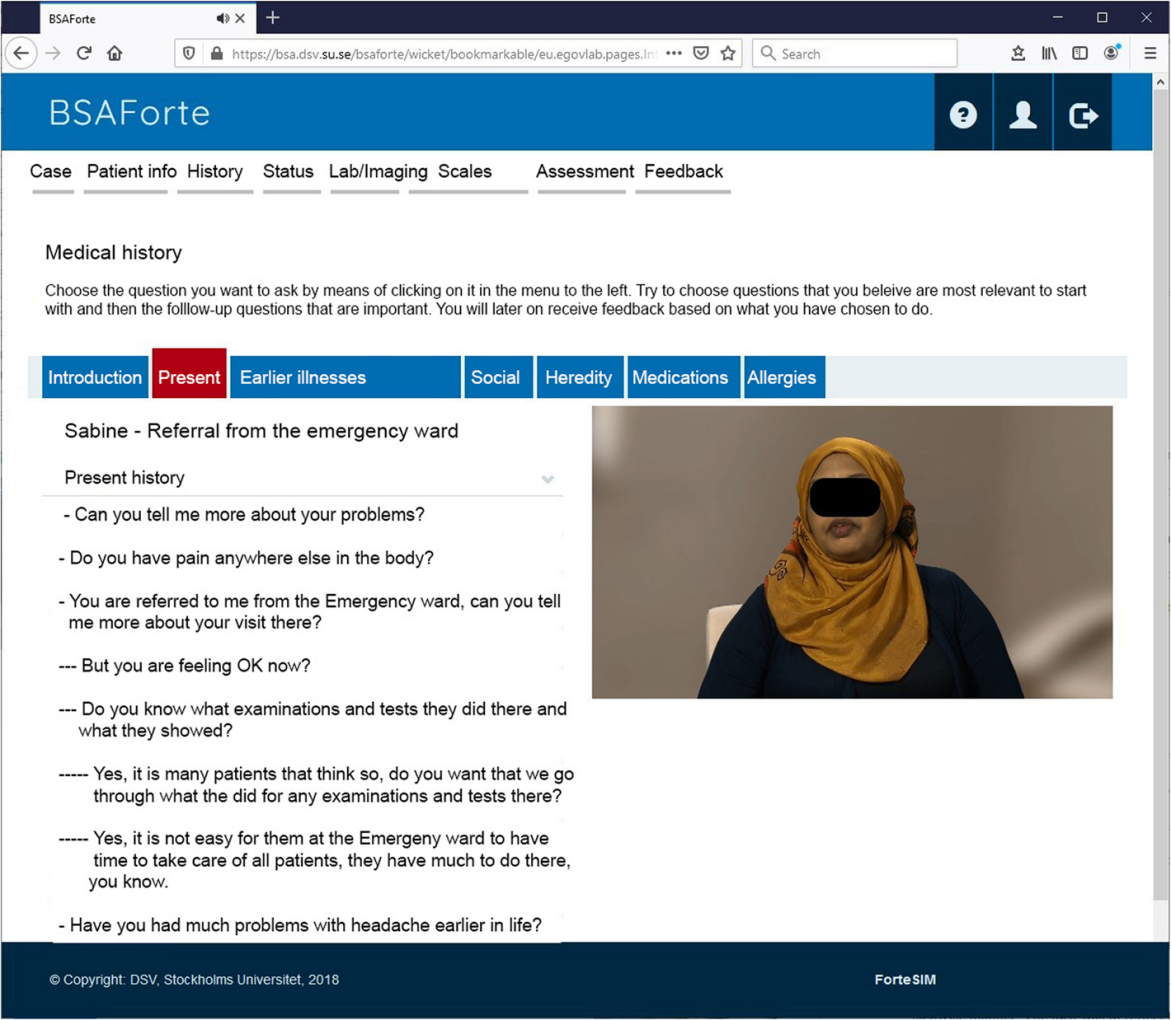
Screenshot showing the layout of the medical history session with one of the virtual patients. The screenshot displays instructions, examples of questions, and the subheadings of the history session

We developed three cases depicting patients with refugee backgrounds and diagnoses, and/or symptoms, commonly addressed in primary care, such as diabetes, musculoskeletal pain, and fatigue (Fig. 1).

In addition to reflecting issues that may arise in intercultural consultations (irrespective of culture), such as language, communication problems, and different views on health and disease, the cases were also designed to illustrate the complexity of the clinical reality facing PCPs. One example of this complexity would be the ability to consider several diagnoses simultaneously and deal with uncertainty, as patients often present with diverse and sometimes vague symptoms.

When working with the VPs, the user was able to take a medical and social history using pre-formulated questions, structured on three levels, with follow-up questions displayed stepwise. The idea was to reflect, as far as possible, the gradual unfolding of information of a real consultation. Many questions were formulated for each case, some “appropriate” and some less so, so the user had to decide which ones to use. The answers from the VP were presented through video clips. The user could also choose among various physical exam procedures, as well as lab and imaging tests. Upon completing the initial encounter, the user was asked to synthesize the information gathered into a free text-based preliminary assessment, and then to make suggestions for a treatment plan.

In addition to interacting with the VP during history-taking, the user was given individualized and automated feedback from the VP, and from a so-called virtual specialist, at the end of each case. This feedback was based on the questions the user had chosen to ask. Each question generated a positive or a negative score, in different feedback categories, in the system. Two different pre-recorded videos with feedback from the VPs were made for each category: a positive and a negative. For example, the positive feedback given from one of the VPs on the category “migration history” states: *“You seemed interested in my background and even though I didn’t feel I could tell you everything today, I felt that you had an interest in me as a person”* (author’s translation), while the negative feedback on the same category was: *“I thought you would ask me a bit more about my background, I think that would have been of importance to address my problems”* (author’s translation). Depending on the summarized score, the user was presented with a more or less “contented” VP, reflecting for example the capability of trust-building. The feedback from the virtual specialist was based on the same scoring system, but was in written form, and focused more on clinical aspects, making suggestions for improvement if needed. The underlying scoring system was not visible to the user.

### Setting and informants

The main rationale for choosing primary care as our setting, was that it is a key element in the Swedish health care system in providing medical care for refugees, covering both somatic and psychiatric spectra [48].

Twelve informants volunteered to participate: eight medical students, three residents, and one specialist in family medicine. Mean years’ work as a PCP were five (range: 4–8). One student was in the eleventh, and final, medical school semester. The other seven were in their fifth semester. There were five women and seven men. The mean estimated time spent on each case was 21 min (range:10–60).

The initial sampling purposively aimed for residents in family medicine, since we believed they, still in training, could make a more valid assessment of the VP as an educational tool. However, specialists wanting to participate were not refused. There were no exclusion criteria. As the interviews indicated that medical students could benefit as well, students on primary care rotation were included at a later stage of the research. Within the respective groups, convenience sampling was applied, since this was deemed the most feasible way to recruit informants [49]. The informants were contacted though three different channels: they were e-mailed through the center for resident education in primary care in Stockholm, invitations were posted on an internet forum for PCPs, and given verbally at various seminars for residents and medical students. Those interested were given further written information and the possibility to ask questions prior to the interview. When written informed consent had been obtained, the informants were given a personal log-in to the VP system, which they could access from a place of their choice. If necessary, they could access the cases repeatedly.

Using the information power model [50], we estimated that 10–15 informants would suffice. This was evaluated throughout the research process, and recruitment was halted when the sample included was deemed to hold sufficient information power (a concept which has been suggested as an alternative to saturation) [50].

### Data collection

A semi-structured interview guide was developed by the research team (Additional file 1). Questions included experiences of working with the VP system, suggestions for improvements and implementation, and accounts of how cultural competence had been addressed so far in their medical education. Phone interviews were then conducted individually by ER. The interviews lasted between 14 and 25 min (mean 17). They were audio-recorded and transcribed verbatim by ER.

One focus group interview was conducted for trustworthiness, where the relevance of the results was confirmed through member-checking [47]. No concepts were changed. This interview took place on campus. It lasted for 43 min and included five additional residents who, after they had had the same VP encounter, were asked to discuss the same topics as the original informants. ER conducted the interview with SE as observer. The informants were also invited to comment on the results. Field notes were made after the interviews and the focus group discussion.

### Data analysis

Data from the interviews was analyzed manually using qualitative content analysis [47]. However, codes and categories were sorted and stored in the NVIVO software. The transcripts were independently read, and coded, by ER and SE, who then compared notes. A few minor differences were reconciled through discussion. Codes were then sorted into preliminary categories by ER. The emerging coding tree was reviewed and discussed among the authors. An overarching theme was obtained through consensus. Throughout the process, we moved from a manifest to a more latent content analysis, in accordance with the method [47]. This allows for interpretation on a more abstract level, while ensuring that the higher-level coding reflected actual data [47]. The analysis was performed in Swedish, being the native language of the research team.

### Ethics

All informants were given verbal and written information on the purpose of the study. Confidentiality was emphasized, as was the right to decline or withdraw at any time without giving reasons. Written informed consent was obtained from all informants. There was no financial reimbursement. Transcripts were anonymized prior to analysis. The virtual patients were portrayed by actors and health care staff with refugee backgrounds, volunteering to participate, thus no patients were at risk. The COREQ guidelines were adhered to (Additional file 2). The study was approved by the Regional Ethical Review Board in Stockholm (2015/1228–31/5, 2016–2308-32 and 2020–01486).

## Results

The analysis generated eight sub-categories, which were sorted into four main categories, generating one common theme: Virtual patients might help improve cultural competence in physicians and medical students by complementing knowledge gained through the informal curriculum.

Training with a VP system, reflecting the clinical reality of primary care, might be useful for physicians and medical students in improving their cultural competence. A complement to what is taught within the informal curriculum, the VP system offers an opportunity to achieve a uniform base for further knowledge.

### Categories and sub-categories

1. Cultural competence is currently obtained via the informal curriculum - VPs constitute a possible complement
  - Cultural competence is not perceived as prioritized in the formal curriculum
  - Training with VPs could compensate for varying preconditions in the clinic

Intercultural issues raised by the VP cases were matters the informants felt had not been prioritized in their medical education. *“I’ve no recollection that we focused on patients with immigrant backgrounds. [It’s] not something I’ve met during my training, more than that I know they exist, so to speak.” (Student informant 8).* Formal learning activities addressing these issues had been scarce if present at all. One exception was learning to work with interpreters, which some could recall had been included in their curriculum. Otherwise, experience-based learning was described as a common way of gaining knowledge in this field. Some informants addressed concerns regarding their dependence on local factors, such as, supervisors’ interest and knowledge, and the sociocultural context of their primary care center. The VP cases could be a way of bridging existing preconditions.” *[It’s] good from an educational point of view that […]loads of students […]can do the same case and gain differing experience, but the same information’s there anyway [… ], it would have been unreasonable for 100 doctors to meet the same patient.” (Student informant11).*

2. The VP-system allows for different levels of pre-existing knowledge
  - VPs might be useful on various educational levels as several aspects of learning are offered
  - User strategies can vary

When discussing possible knowledge gained from the VP system, the informants highlighted various learning aspects, maybe partly reflecting their level of medical education. For example, residents mentioned the opportunity for reflection. *“During resident training it’s more of a reflection on how I normally do things [ … ]and you can’t really see this during medical training [ …], then it’s more about gaining knowledge of these issues for example. But here it’s more how one delves deeper into how one works; I think this is a good thing and very valuable during resident training.” (Physician informant 3).* Students, on the other hand, appreciated other aspects, such as the possibility to practice writing a preliminary assessment of the case. In general, residents felt it would probably be beneficial to introduce VP cases earlier in the education process: medical students could, for example, prepare for future patient contact through training with VPs. On the other hand, some students expressed that having more medical knowledge might help one focus more on the intercultural aspects. Strategies for working with the cases varied, some describing an approach mirroring what they would do in real life, while others applied a freer approach. The latter involved, for example, clicking on every question available, just to see the response. Another example was to ask the VP questions they normally would avoid, feeling that it was okay to make mistakes and not having to perform.

3. Appreciated features of the VP system were authenticity and interactivity
  - Realistic case-content reflects the complexity of primary care
  - The possibility to interact with the VP offers a different way of learning

The learners highlighted two features of the VPs as particularly useful: the authenticity and the interactivity. The content of the VP cases was described as realistic. Residents working with patients with refugee backgrounds on a regular basis, confirmed that issues incorporated in the VP cases, reflected those they met in everyday practice.” *I think you can learn a lot from this and since I often work with patients with refugee backgrounds, I recognize loads of this and have, sort of, got the knack of taking a history and, kind of, moving on.” (Physician informant 9).* Students especially appreciated how the VP system could create a case-based context for gaining intercultural competence.*”It’s easy to say that in some cultures they do things like this or like that, but I think it’s an extremely good way of getting examples of this in context.” (Student informant 10).* In addition to the intercultural aspects, the VPs authentically illustrated the complexity of family medicine in general; medical and social issues are often intertwined, and there is often more than one way to approach a problem.*“That there isn’t a genuine clinical answer, that’s the reality, its seldom like ‘yes, now we know what it is, now you can go home.”(Physician informant 1).*

The interactive part of the VP system was also appreciated by the informants, with the feedback from the VPs being emphasized as especially important for retaining knowledge. The informants described how being addressed directly and verbally by the VP, even though through pre-recorded videos, could evoke an emotional response which they believed could facilitate learning. *“Now at any rate I have before my eyes the face of [the VP] who said [ …], and it sticks in my mind in a different way I think.” (Student informant 11).*

4. There is room for improvement, but the VP system seems relevant in learning cultural competence
  - The VP system could, with some adjustments, be a useful addition to cultural competence training
  - Combining the VP system with a discussion-based learning activity might enhance knowledge retention

In general, the informants found VPs suitable for future cultural competence training in a primary care context. However, to optimize learning, suggestions for improvements to the VP system were made. These focused mainly on the history-taking section, where a more authentic feel to the interaction was wanted. For example, some felt they wanted to validate the patient to a greater extent than the program allowed for. *“You want to be able to say ‘I realize it’s hard for you and things like that.” (Student informant 5).* Some informants also wished for a possibility to formulate text freely. Others, however, expressed that they found the pre-formulated questions useful, as they viewed them as suggestions for what to ask. There was also disagreement among the informants on whether the feedback they received from the patient was “fair”, given their performance.

Pedagogical methods to facilitate intercultural learning from the VP system were discussed, but the data was not conclusive. As the cases gave rise to numerous thoughts and questions, there was a general wish to be able to discuss them further, either with other students or in a teacher-led seminar. Some also suggested that using the VP system should be mandatory, instead of a voluntary add-on.*” If it’s voluntary it’s easy to skip it, I’m afraid … ‘yes but I’ve got a test I must study for’ and then you don’t do it properly. But if you have it in a group, in a seminar, it’s much better.” (Student informant 4).*

## Discussion

### Statement of principal findings

The study sought to explore a learner perspective on the educational use of a virtual patient (VP) system, designed to contribute to cultural competence training, in a primary care context. Our findings show that the informants, both on pre- and post-graduate levels, found it useful as a complement to clinical training. The VP system was found suitable as an introduction to intercultural consultations, as well as for stimulating reflection. User strategies and learning points varied among the informants, maybe partly due to their different educational levels. In general, the importance of authenticity and the possibility to interact with the VP, were highlighted as features likely to enhance learning. The informants’ views on future educational contexts where the VP system could be useful, pointed, in general, towards some form of discussion-based learning activity.

### Findings in relation to previous work

Our VP system may contribute to cultural competence training in a primary care context, as the informants described cultural competence as an area of knowledge often taught informally, and in general they felt it had been somewhat neglected during medical training. The residents could not clearly recall whether cultural competence had been taught during medical school; likewise, the students interviewed felt that it was not an area prioritized in their current curriculum. This tallies with previous studies, indicating that residents in family medicine learn cultural competence through the informal curriculum [9, 19]. Although most medical schools today include the topic in the formal curriculum, it is often taught cursorily and students have difficulties applying what they have been taught [18, 51]. Informal learning is not in itself negative, but the learner risks becoming over-reliant on “ad hoc” learning, having to rely on patient encounters as triggers for learning [20]. Previous VP studies have highlighted the possibility of using VPs to ensure that diagnoses common in the population, but not frequent at, for example, highly-specialized teaching hospitals, are covered; this is also in line with trying to avoid being over-reliant on “ad hoc” learning [52]. In terms of gaining cultural competence in primary care, triggers for learning may or may not occur depending on, for example, the socioeconomic context of the area the health care center serves. Introducing VPs might be one way of compensating for this, both by ensuring that educational material covering core issues is available for all, and by in offering opportunities for more organized learning activities in the clinic. An additional benefit might be improved knowledge retention, since learning taking place through some form of planned activity is better than reactive learning, where knowledge is considered at best a by-product of the task in hand [53, 54].

Using VPs as complementary learning tools to what is taught within the informal curriculum, such as cultural competence, requires VP systems to meet educational needs on different levels. Satisfyingly, our VP system was in general perceived as useful, regardless of the level of prior knowledge. Some residents described how working with the VPs became a trigger for reflection on, for example, cultural differences and one’s own behavior in intercultural consultations – an important component of being able to work in a culturally competent manner. Being reminded of issues regularly occurring in the clinic, but seldom reflected on, has also previously been highlighted as an advantage of VP training [28]. Both residents and students thought VPs could be useful for introducing the topic in medical school before clinical rotations. Other learning points were also emphasized by the students, for example, training basic skills, such as, writing a preliminary assessment of the case. This finding illustrates how the use of VPs not necessarily follows only the designer’s intention, but instead, is likely to be more diverse.

Thus, what is gained from the VP system might vary with the user’s educational level, even though there was agreement that authenticity and interactivity were crucial elements for awakening and maintaining an interest in the cases, and consequently for gaining knowledge. This tallies with previous research, highlighting the importance of relevance and authenticity in evoking engagement and promoting learning [55, 56]. Several factors contributing to the authenticity of VPs have been identified, such as the type of case, realistic dialog, and acting [26]. Our informants emphasized the importance of the content of the case in creating a feeling of authenticity and relevance, rather than e.g. the quality of the acting or the interface. Ambiguity and uncertainty are feelings often present in primary care consultations [57, 58]; accordingly, these were elements we tried to incorporate into the VP system to increase authenticity. This was also noted and appreciated, primarily by the residents, likely a reflection of their greater clinical experience. Some informants saw cultural competence as an abstract concept and appreciated the relatively realistic framework the VP system provided to illustrate why it is important, and how to apply it, in their everyday work. We have not seen this finding discussed to any extent in other VP studies, but it is, nonetheless, a possible significant advantage.

The possibility to interact with the VP is also likely to contribute to a feeling of authenticity. Even though the informants wished for a more realistic interaction in the history-taking section, they appreciated the patient feedback as an opportunity to share an unfiltered patient perspective, something they did not get in real-life consultations. Interestingly the views on whether the feedback was ‘fair’ differed. This did not seem to be a product of educational level, as perceived fairness versus unfairness was found among both residents and students. Instead the following three aspects seem more plausible to consider. First, the various perceptions might reflect variations in the ability to evaluate one’s own knowledge or skills. Secondly, the variation in the estimated time spent on the cases might matter, since choosing questions more carefully is likely to generate a more favorable score. Finally, the programming of the scoring system might also play a part: if for example some questions are given too much weight, this could result in unfair feedback. However, one might expect a sense of “unfairness” to be more pervading, were that the case. To clarify whether this is an area for improvement in our VP system, further exploration is required. For future improvement, considering a stepwise feedback-system may also be of interest. In general, making greater use of the possibilities of taking the patient perspective into account, is an interesting area for future VP studies, this has also been suggested previously [55].

Future studies are also required of what pedagogical methods to use to facilitate learning cultural competence from VPs, taking contextual factors into account. Exploring the validity and reliability of the VPs in a structured manner would also be of interest. Other areas to consider for future use of VPs would be medical professionalism and dealing with uncertainty, which today, in primary care, just like cultural competence, seem to be taught largely through the informal curriculum [19].

### Limitations

We considered the main limitation of the study to be the use of convenience sampling, even though it is a common method of sampling in qualitative studies. To increase credibility, we tried to ensure a representative sample, with high information power, by including both students and physicians, with varying experience of intercultural consultations. The result of the analysis was confirmed through member-checking, which also contributed to the credibility. Conducting the interviews by phone is not optimal, but given the limited amount of time available, this was deemed the most feasible option.

The interviews were performed by ER, who was also involved in developing the VP system. Although this was not conveyed in detail to the informants, they were informed that ER was part of the research team. Thus, the position of the interviewer could have been a factor hindering the informants from speaking freely about negative aspects of the system out of social desirability. At the same time, we presumed that using an external interviewer, without deeper knowledge of the VP system, would likely generate less rich data. Instead we chose to encourage the informants to criticize the system, emphasizing that it would help us improve it.

As for transferability, it is for the reader to evaluate. However, we suggest that although the study was set in a Swedish context, the results are likely to be transferable to similar VP systems, being evaluated in comparable primary care settings. Including cultural competence in the informal curriculum is not exclusive to Sweden and the wish for increased knowledge in the area is also widespread.

## Conclusions

VPs could be a suitable tool for cultural competence training in a primary care context, one advantage being decreased reliance on what ‘happens to be taught’ within the informal curriculum. The flexibility of the VP system seems to allow learners to use it for different purposes, depending on prior knowledge, which permits possible implementation on various educational levels. While increased cultural competence in health care has been called for, students and physicians alike sometimes find it abstract and consequently difficult to implement. Concretizing the concept through training with VPs might be one way forward.

## Supplementary Information

**Additional file 1.** The interview guide developed by the research team.

**Additional file 2.** COREQ checklist.

## Data Availability

Transcripts of the interviews analyzed are not publicly available due to the risk of compromising individual privacy, but data is available from the corresponding author on reasonable request. The questionnaire used is submitted as an additional file.

## Abbreviations

PCP: Primary care physician
VP: Virtual patient
