# Supplementary material for: Virtual patients reflecting the clinical reality of primary care – a useful tool to improve cultural competence
Source: BMC Med Educ. 2021 May 11;21:270. doi: 10.1186/s12909-021-02701-z (PMC8112065; doi:10.1186/s12909-021-02701-z)
Supplement: Supplementary file 1 — Additional file 1. The interview guide developed by the research team. [file 12909_2021_2701_MOESM1_ESM.pdf]

Additional file 1. Interview guide

What is your general experience of this type of interactive cases?

What are your thoughts on these cases and how to improve them?

Follow-up: Give examples

Estimated time spent on each case?

In what learning context/format would they be suitable in your opinion?

Follow-up: Why/give examples

What would be an appropriate target group for training with these cases?

Follow-up: Residents, interns and/or medical students? Why/why not?

Considering the cases portrayed, do you think any additional information would have been beneficial in increasing your intercultural knowledge/understanding?

Follow-up: If so, give examples

What and *how* have you learned about intercultural consultations, and more specifically about consultations with refugee patients, so far during your medical training?

Follow-up: If anything, what has been missing?

Could you give an example of something (if anything) you learned when going through these cases that you might apply/think about in your next consultation with this group of patients?

Other questions or comments?

What are your thoughts on the interview?
